# Ultra‐Low Concentration Electrolyte Enabling LiF‐Rich SEI and Dense Plating/Stripping Processes for Lithium Metal Batteries

**DOI:** 10.1002/advs.202203216

**Published:** 2022-08-17

**Authors:** Ting Chen, Jinhai You, Rong Li, Haoyu Li, Yuan Wang, Chen Wu, Yan Sun, Liu Yang, Zhengcheng Ye, Benhe Zhong, Zhenguo Wu, Xiaodong Guo

**Affiliations:** ^1^ Department of Chemical Engineering Sichuan University Chengdu 610065 P. R. China; ^2^ Laboratory for Soft Matter and Biophysics Department of Physics and Astronomy KU Leuven Leuven 3001 Belgium; ^3^ School of Mechanical Engineering Chengdu University Chengdu 610106 P. R. China; ^4^ School of Materials Science and Engineering Henan Normal University Xinxiang Henan 453007 P. R. China; ^5^ Institute for Advanced Study Chengdu University Chengdu 610106 P. R. China

**Keywords:** dense plating/stripping process, electrolyte concentration, lithium metal batteries, pouch cell, solid electrolyte interphase

## Abstract

The interface structure of the electrode is closely related to the electrochemical performance of lithium‐metal batteries (LMBs). In particular, a high‐quality solid electrode interface (SEI) and uniform, dense lithium plating/stripping processes play a key role in achieving stable LMBs. Herein, a LiF‐rich SEI and a uniform and dense plating/stripping process of the electrolyte by reducing the electrolyte concentration without changing the solvation structure, thereby avoiding the high cost and poor wetting properties of high‐concentration electrolytes are achieved. The ultra‐low concentration electrolyte with an unchanged Li^+^ solvation structure can restrain the inhomogeneous diffusion flux of Li^+^, thereby achieving more uniform lithium deposition and stripping processes while maintaining a LiF‐rich SEI. The LiIICu battery with this electrolyte exhibits enhanced cycling stability for 1000 cycles with a coulombic efficiency of 99% at 1 mA cm^–2^ and 1 mAh cm^–2^. For the LiIILiFePO_4_ pouch cell, the capacity retention values at 0.5 and 1 C are 98.6% and 91.4%, respectively. This study offers a new perspective for the commercial application of low‐cost electrolytes with ultra‐low concentrations and high concentration effects.

## Graphical abstract

In view of the high‐concentration effects that can be achieved by high‐concentration electrolytes, including excellent electrochemical performance, LiF‐rich SEI, and dense lithium plating/stripping process, an ultra‐low concentration electrolyte with high‐concentration effects is designed using inert diluents. The electrolyte achieves the dual goals of maintaining a high concentration effect and reducing costs, showing great application value.

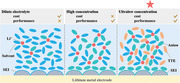

## 1 Introduction

Lithium metal has been hailed as the “Holy Grail” anode for Li‐metal batteries (LMBs) owing to its extremely high specific capacity (3680 mAh g^−1^) and very low electrochemical potential (−3.04 V vs the standard hydrogen electrode).^[^ ^1^ ^]^ Renewed interest in rechargeable LMBs has been spurred by the growing global energy demand.^[^ ^2^ ^]^ However, LMBs face substantial challenges, including lithium dendrites and unsatisfactory coulombic efficiencies (CEs).^[^ ^3^ ^]^ These drawbacks are related to the easily destroyed but unrepairable solid electrolyte interphase (SEI) film formed through the reaction between the liquid electrolytes and Li metal.^[^ ^4^ ^]^ Therefore, it is necessary to improve the structure of the solvated layer of lithium ions by regulating the composition of the electrolyte, such as the lithium salt, solvent, and additive, to form a high‐quality SEI interface in situ, which will ultimately improve the cycle performance of the lithium metal anode.^[^ ^5^, ^6^ ^]^ To achieve an LMB system with a high CE, the electrolyte must be able to rapidly form a stable SEI with high mechanical strength and good ion conductivity that can effectively isolate the contact between the remaining electrolyte and lithium metal. Furthermore, an electrolyte with excellent electrochemical performance is conducive to achieving dense lithium deposition, reducing dendrite growth, and improving battery safety.

Recent studies have shown good compatibility between ether‐based electrolytes and lithium metal.^[^ ^7^ ^]^ Although the CE of lithium metal in this type of electrolyte can exceed 90%, it does not have sufficient stability to support real applications.^[^ ^8^ ^]^ It is believed that increasing the concentration of the electrolyte can provide the following advantages: (1) regulation of the solvation behavior of Li^+^ to obtain an SEI interface with high chemical stability that is rich in inorganic substances such as LiF and has excellent ion conductivity;^[^ ^9^, ^10^ ^]^ and (2) inhibition of the growth of lithium dendrites and delay of the corrosion of the collector by the electrolyte.^[^ ^10^ ^]^ Although carbonate ester‐based electrolytes have a high dielectric constant and anodic stability, they always result in low CE. Importantly, when the concentration of the ester electrolyte is increased, it is difficult for the high‐concentration ester‐based electrolyte to maintain a liquid state at room temperature.^[^ ^11^ ^]^ Therefore, a high‐concentration ether‐based electrolyte can improve the interfacial stability of lithium metal anodes.^[^ ^12^ ^]^ However, high‐concentration ether‐based electrolytes have the shortcomings of poor wettability, high viscosity, and high cost owing to the extensive use of expensive lithium salts.^[^ ^13^ ^]^ To overcome these challenges and retain the excellent characteristics of high‐concentration electrolytes (HCEs), some researchers have selected reasonable diluents (including bis(2,2,2trifluoroethyl) ether (BTFE), 1,1,2,2‐tetrafluoroethyl‐2,2,2‐trifluoroethyl ether (TFETFE), and 1,1,2,2‐tetrafluoroethyl‐2,2,3,3‐tetrafluoropropyl ether (TTE)) as cosolvents to form local high‐concentration electrolytes.^[^ ^5^, ^14^ ^]^ Significant progress has been made in research on high‐concentration ether electrolytes in LMBs,^[^ ^15^ ^]^ but previous studies have paid little attention to the effect of further dilution of the electrolyte on the interface of the lithium anode and the excellent electrochemical performance of pouch cells.

In this study, a new ether electrolyte system was designed. Tetrahydrofuran (THF), which is inexpensive, readily available, and has low viscosity, was used as the solvent, while lithium difluorosulfonimide (LiFSI) with high ionic conductivity was used as the lithium salt. Table S1 (Supporting Information) lists the electrolyte formulations used in this study. Considering the cost and viscosity, D5 was diluted to H1 with TTE; the electrolyte solutions were designed by combining theoretical simulations with experimental results. The results show that contact ion pairs and aggregation ion pairs are only separated by the diluent TTE into island aggregates, while the solvation structure of lithium ions in the electrolyte is not changed. The LiIICu half‐cells matched with H1 are stable for 1000 cycles and can maintain a CE of ≈99% at 1 mA cm^–2^ and 1 mAh cm^–2^, showing excellent electrochemical stability. In the system, lithium metal is matched with the LiFePO_4_ cathode, and the coin cells exhibit excellent cycling performance without capacity attenuation for 100 cycles under a cathode loading of up to 1.79 mAh cm^–2^. For ultra‐low concentration electrolyte H2, a pouch cell prepared using a LiFePO_4_ cathode (2.65 mAh cm^–2^) and 50 µm lithium foils as the anode is measured at 0.5 and 1 C. The capacity retentions of the pouch cells at these current densities are 98.6% and 91.4%, respectively. Therefore, this method is meaningful for designing a commercial electrolyte with both excellent electrochemical performance and low cost for LMBs. Thus, this electrolyte system has real application value.

## 2 Results and Discussion

**Figure 1:**
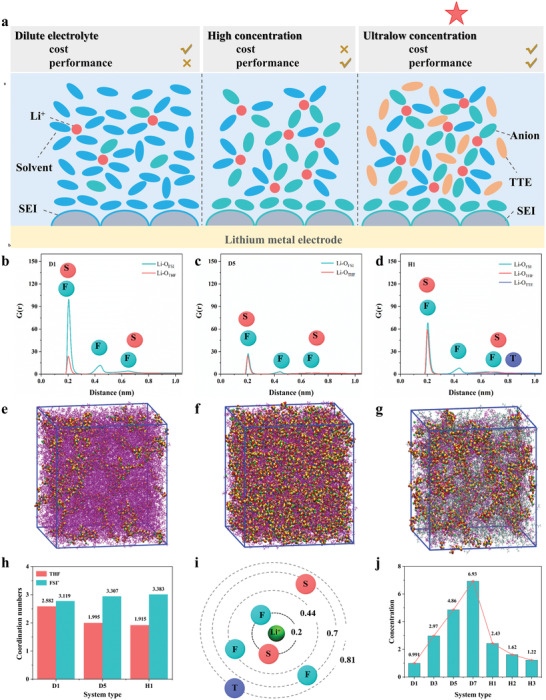
a) Design models of different electrolytic liquid systems. b–d) Radial distribution functions of dilute electrolyte (D1), HCE (D5), and LHCE (H1); S, F, and T denote the solvent, anion, and diluent, respectively. e–g) Molecular dynamics (MD) simulation results for the dilute electrolyte (D1), HCE (D5), and LHCE (H1). h) Coordination numbers of Li^+^ with solvent molecules (THF) and anions (FSI^–^) in electrolytes D1, D5, and H1. i) The circles in (b)–(d) and (i) indicate the coordination between the specific item and Li^+^. j) Concentrations of all of the electrolytes designed in this study.

**Figure** **1**a illustrates the concept of this study. The ultra‐low concentration electrolytes in this study were all further diluted and optimized based on D5. Molecular dynamics (MD) simulations were performed to study the coordination of Li^+^ ions in different electrolytes (Table S1 and Figure S1, Supporting Information). The radial distribution schemes of Li and O atoms in the THF solvent, FSI^–^ anion, and TTE diluent based on the MD simulation trajectories are shown in Figure 1b–d. The results show that all of the electrolyte systems exhibit two sharp peaks at 0.2 nm, corresponding to Li–O in THF and Li–O in FSI^–^. Optical images of the MD simulation diagrams are shown in Figure 1e–g, revealing the states of the molecules in each solution system. We found that the molecular state of H1 is closer to that of D5, which is very different from that of the dilute electrolyte D1. However, owing to the addition of the TTE diluent, the molecular layer in H1 is divided into multiple parts by the TTE. Figure 1h shows the coordination numbers of Li^+^ and the solvent molecules (THF) and anions (FSI^–^) in each electrolyte. The results show that as the electrolyte concentration increases, the number of Li^+^ ions coordinated with THF gradually decreases, but the number of Li^+^ ions coordinated with salt anions increases. In locally HCE systems such as H1, the coordination number between TTE and Li^+^ is 0. Based on the radial distribution function between the atoms, the molecular orbital distributions are summarized in Figure 1i. The results show that in D5 and H1, anions gradually enter the interior of the Li^+^ solvation layer, and the addition of the diluent TTE does not change the solvation structure of the high‐concentration electrolyte. All of the lithium ions in the electrolyte are surrounded by THF solvent molecules and FSI^–^ anions in the first coordination shell. This is caused by the strong interaction between Li^+^ and THF/FSI^–^, and these results are similar to those of other studies.^[^ ^16^, ^17^ ^]^ At the same time, some studies have shown that although the inert diluent does not have a strong impact on the solvation structure of the HCE liquid phase, the physicochemical properties at the electrolyte interface are not necessarily similar,^[^ ^18^ ^]^ and thus it is impossible to ensure that the electrochemical properties of the electrode in these two types of electrolytes are completely consistent.^[^ ^19^ ^]^ In this study, a series of electrolytes with different concentrations are designed according to the above results, as shown in Figure 1j.

To obtain an electrolyte with an ultra‐low concentration and excellent performance, a series of experimental operations and theoretical calculations are required. First, by comparing the CE of LiIICu cells in different electrolytes, we investigate the effect of the electrolyte concentration on the deposition–stripping behavior and cycle stability of lithium metal to select the electrolyte with the optimal concentration on the premise of controlling the cost. Figure S2 (Supporting Information) shows the CE of the LMB in the electrolyte at four different concentrations. By changing the charge–discharge current density and areal capacity, we can determine that D5 is the best high‐concentration electrolyte system. As shown in Figure S3 (Supporting Information), when the lithium metal is cycled for 10 cycles at 0.5 mA cm^–2^ and 1 mAh cm^–2^ in D5, the Li deposition layer is compact and homogenous without the formation of detrimental wispy Li dendrites. At the same time, the thickness of this layer is only 12.61 µm, which further confirms that D5 is the optimal electrolyte system. This result shows that increasing the electrolyte concentration can effectively reduce the deposition thickness of lithium metal and increase its density.

**Figure 2:**
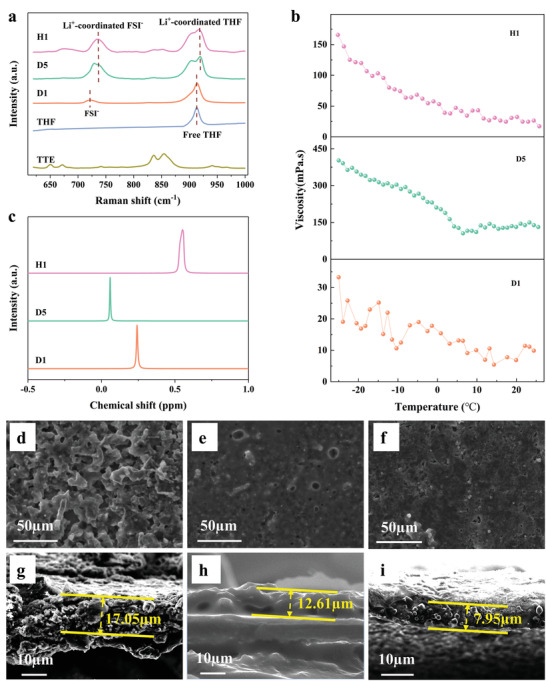
a) Raman spectra of TTE, THF, D1, D5, and H1 at 25 °C. b) Viscosity of D1, D5, and H1 at temperatures of −25 to 25 °C. c) Comparison of the ^7^Li‐NMR results for the three electrolytes at 25 °C. Top‐view and cross‐section SEM images of the Li deposition morphologies in different electrolytes: d,g) D1; e,h) D5; f,i) H1.

A rapid comparison of the physical and chemical properties of the three types of electrolytes was carried out using Raman spectroscopy, ^7^Li‐NMR, and viscosity analysis. First, we investigated the progression of the Raman spectra of the electrolytes, as shown in **Figure** **2**a. With an increase in the LiFSI salt concentration to form D5 in pure THF, the free THF (O–CH_3_ stretching vibration band at ≈913 cm^–1^) gradually diminishes to form Li^+^‐coordinated THF (≈920 cm^–1^).^[^ ^20^ ^]^ With dilution by TTE, the Li^+^‐coordinated THF solvation structure is well preserved, and the vibration band of TTE at 800–880 cm^–1^ does not change in H1. Importantly, TTE barely weakens the association between Li^+^ cations and FSI^–^ anions, as evidenced by the Li^+^‐coordinated FSI^–^ Raman band (710–780 cm^–1^).^[^ ^21^ ^]^ This conclusion is consistent with the MD simulation results.

Further confirming the above conclusion, the chemical shifts of D5 and H1 both widen compared to the dilute electrolyte (D1) in the ^7^Li NMR characterization results of the different electrolytes at 25 °C (Figure 2c). However, compared to D5, the chemical shifts of H1 shift toward the low field, indicating that the TTE diluent does not disturb the solvated layer structure of Li^+^. Thus, it is likely that the addition of TTE only separates large ion clusters in the D5 electrolyte, which echoes the MD simulation results. The Fourier transform infrared spectroscopy (FT‐IR) characterization results (Figure S4, Supporting Information) show that as the electrolyte concentration increases, the wavelength of the corresponding characteristic groups moves to a higher wavenumber, i.e., a “blue shift.” This indicates that the interactions of both the solvent and anion with Li^+^ become stronger,^[^ ^22^ ^]^ which is also verified by the ^7^Li‐NMR characterization results.

Importantly, the viscosities of the three electrolytes (Figure 2b) show that the addition of the TTE diluent can significantly reduce the viscosity of the high‐concentration electrolyte (D5), and the viscosity of H1 is approximately the same as that of dilute electrolyte D1. The wettability of the Cu foil and separator was measured using a contact angle goniometer. The results (Figures S5 and S6, Supporting Information) confirm that regardless of the Cu foil or separator, the wettability of the dilute electrolyte is good, whereas the wettability of D5 is poor. However, the wettability of H1 formed after the addition of TTE is greatly improved, and is even better than that of the dilute electrolyte, which further verifies our expectations.

To test the electrochemical performance of the above electrolyte systems, we applied them to LiIICu batteries to observe the deposition morphology and thickness under the condition of equal deposition and stripping. As shown in Figure 2d,g, the lithium foil exhibits an irregular loose structure in the dilute electrolyte (D1); the deposition thickness is 17.05 µm, and the initial coulombic efficiency (ICE) of lithium is 95.48% (Figure S7, Supporting Information). When the electrolyte concentration is increased to D5, the lithium deposition layer is more dense, the thickness of the deposition layer is reduced to 12.61 µm (Figure 2h), and the ICE also increases to 97.48%. These results indicate that increasing the electrolyte concentration can effectively inhibit excessive side reactions between the lithium metal and solvent while improving the ICE of the battery. When inert TTE diluent is added, the deposited layer becomes denser, and the thickness is greatly reduced to 7.95 µm (Figure 2i). The ICE also increases to 99.30%. The enhancement of the CE is consistent with the improvement in the deposition layer observed using scanning electron microscopy (SEM).

**Figure 3:**
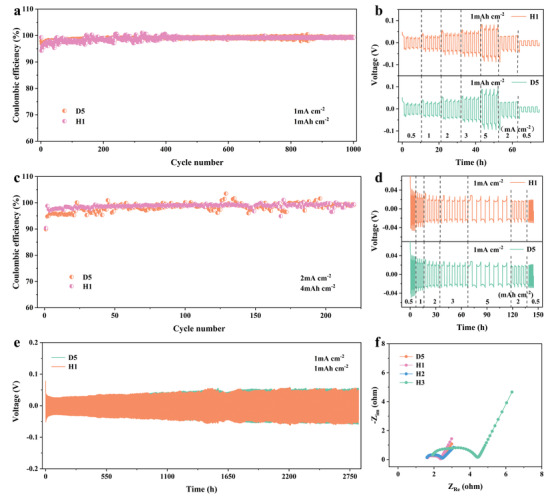
CE of LiIICu cells with a) 1 mAh cm^–2^ at 1 mA cm^–2^ and c) 4 mAh cm^–2^ at 2 mA cm^–2^ in D5 and H1. Rate performance of LiIILi symmetrical cells with b) a fixed capacity of 1 mAh cm^–2^ and d) fixed current density of 1 mA cm^–2^ in H1 and D5. e) Excellent cycling performance of LiIILi symmetrical cells in H1 and D5. f) Evolution of the impedance spectra of the LiIILi symmetric cells after 30 cycles in the D5 and H1 electrolytes.

To better evaluate the influence of these solvents on lithium metal, it is necessary to evaluate the CE of LiIICu batteries and the cycling stability of symmetrical cells in D5 and H1, which can mimic the charge–discharge process in full cells. The CE is a key parameter for measuring the sustainability of lithium metal anodes.^[^ ^23^ ^]^ The CE needs to be compared under different current densities and areal capacities in the electrolytes. Importantly, we also evaluate the Li CE in D5 and H1 at a high current density (2 mA cm^–2^) and high surface capacity (4 mAh cm^–2^). In **Figure** **3**a, the Li CEs during cycling in two different electrolytes are compared. In the concentrated D5 electrolyte, the Li CE during cycling at 1 mAh cm^–2^ and 1 mA cm^–2^ increases to ≈99.3% and remains stable for over 1000 cycles. In the H1 electrolyte, the battery can also achieve a CE of ≈99.4% for more than 1000 cycles under the same conditions. In addition, Figure S8a,b (Supporting Information) shows the voltage profiles of the LiIICu cells at 1 mA cm^–2^ and 1 mAh cm^–2^. The overpotential in D5 at the 100th cycle is only 56 mV and increases to 98 mV at the 1000th cycle; however, the overpotential of the battery in H1 is 86 mV at the 100th cycle. With an increase in the number of cycles, the overpotential gradually increases. When the battery is at the 1000th cycle, the overpotential of the cell is 187 mV, which suggests it is in the initial stage of cycling under the conditions of low current and low areal capacity. When the cells are further cycled at a higher current density (2 mA cm^–2^) and capacity (4 mAh cm^–2^) (Figure 3c), the CE exhibits much smaller fluctuations for H1, while the CE of both electrolytes can reach ≈98.7% overall. Furthermore, D5 and H1 show similar voltage polarization (Figure S8c,d, Supporting Information) as the areal capacity increases, indicating that the Li anode in H1 demonstrates a more stable plating/stripping process. These results show that the addition of TTE diluent can maintain and surpass the high CE of the original high‐concentration electrolyte.

As shown in Figure 3b, the rate performance of the symmetrical cells is tested at a fixed capacity of 1 mAh cm^–2^. The Li anode in electrolyte H1 delivers stable voltage hysteresis changes of 40, 50, 64, 80, and 114 mV at rates of 0.5, 1, 2, 3, and 5 mA cm^–2^, respectively, which are slightly lower than those in electrolyte D5. The rate performance of a symmetrical cell at a fixed current density is shown in Figure 3d. Additionally, at 1 mA cm^–2^ and 1 mAh cm^–2^, LiIILi cells using the H1 electrolyte exhibit excellent cycling stability, with nearly constant voltage hysteresis over 2800 h, as shown in Figure 3e. Figure S9 (Supporting Information) demonstrates that when the current density and capacity are further raised to 2 mA cm^–2^ and 4 mAh cm^–2^, respectively, the symmetrical cells with the two types of electrolytes still exhibit excellent cycle stability (over 1400 h). The D5 and H1 electrolytes presented in this study show outstanding compatibility with lithium metal, indicating that the excellent electrochemical performance of the HCE is not weakened by the addition of the TTE diluent, thus demonstrating the application prospects for the LMB full cell.

**Figure 4:**
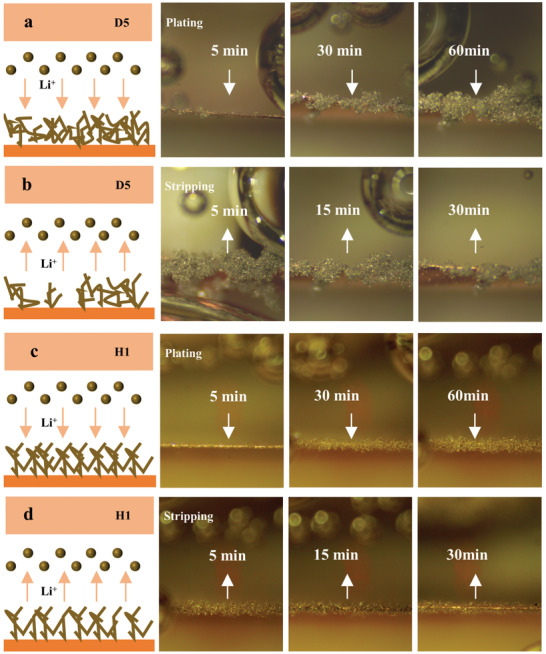
In situ digital photographs of LiIICu batteries in a,b) D5 and c,d) H1 electrolytes during the lithium plating and stripping processes.

**Figure** **4** shows the plating and stripping processes of Li metal in the two electrolytes. Comparing the plating processes (Figure 4a,c), we find that Li metal first deposits relatively unevenly in spots in the higher‐concentration D5, while the deposition morphology of Li metal in the lower‐concentration H1 is more uniform. As the plating process progresses, the Li metal deposits in D5 begin to connect and grow at the previously prioritized nucleation locations. Although the uniformity is not as good as that of H1, the overall lithium deposition is relatively dense. In contrast, in the lithium stripping process (Figure 4b–d), the Li metal in D5 first dissolves from the junction of the lithium nucleation site. We speculate that the lower concentration of H1 results in a more uniform Li‐ion flux owing to the lower electrolyte concentration gradient.

**Figure 5:**
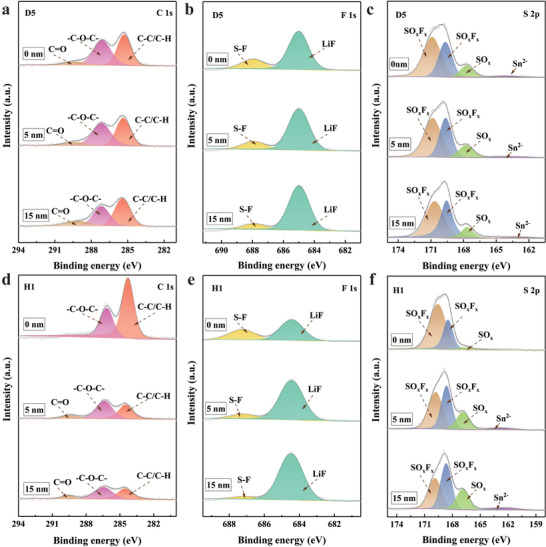
a,d) C 1s; b,e) F 1s; and c,f) S 2p spectra at different depths of the Li metal anode in H1and D5 after 50 cycles.

To further understand the high CE obtained in D5 and H1, X‐ray photoelectron spectroscopy (XPS) equipped with argon‐ion sputtering was used to analyze the SEI component distribution of several elements formed on the Li anode in different liquid electrolytes. The SEIs of the plated Li on the Cu foils after 50 cycles at 1 mA cm^–2^ and 1 mAh cm^–2^ were characterized, as shown in **Figure** **5**. The C 1s spectra (Figure 5a,d) show that the carbonate compositions in the SEI on the Li metal in D5 and H1 are very similar, but the carbonate content in H1 is lower than that in D5. In addition, the F 1s spectra (Figure 5b,e) demonstrate that the SEIs formed on Li metal in D5 and H1 contain large amounts of LiF. Compared with the absence of the S‐F peak in D5 and H1, this result indicates that almost all the F in H1 and D5 originates from the decomposition of the salt anion FSI^–^. Because the S in the SEI only exists as LiFSI, it is necessary to measure the S 2p of the SEI. As shown in Figure 5c–f, more sulfide is present in D5 and H1, which indicates that a large amount of salt anions react and decompose in the SEIs of these two electrolytes. Meanwhile, the distributions of S in H1 and D5 are basically the same. Based on these results, we can conclude that the SEI in these two electrolytes is mainly composed of organic and inorganic compound structures due to partial solvents and salts participating in decomposition during the plating and stripping processes, while FSI^–^ anions mainly react violently with Li^+^ to form an LIF‐rich inorganic SEI layer.^[^ ^24^ ^]^

LiF has been shown to effectively inhibit the growth of lithium dendrites and accelerate the surface diffusion rate of ions owing to its high interfacial energy.^[^ ^25^ ^]^ Considering the similar linear scanning voltammetry (LSV) profiles of D5 and H1 (Figure S10, Supporting Information), the results indicate that the FSI^–^ anions coordinated with Li^+^ preferentially decompose upon contact with metallic Li during Li plating, leading to passivation of the Li metal by the LiF‐rich SEI layer. Importantly, through the spectral analysis of the three elements, we can conclude that the composition and distribution of the SEI on the Li anode in H1 are approximately consistent with those in D5, which confirms that both electrolytes provide a high CE and further indicates that the electrochemical performance advantage induced by the high‐concentration electrolyte is not weakened by the addition of TTE.

**Figure 6:**
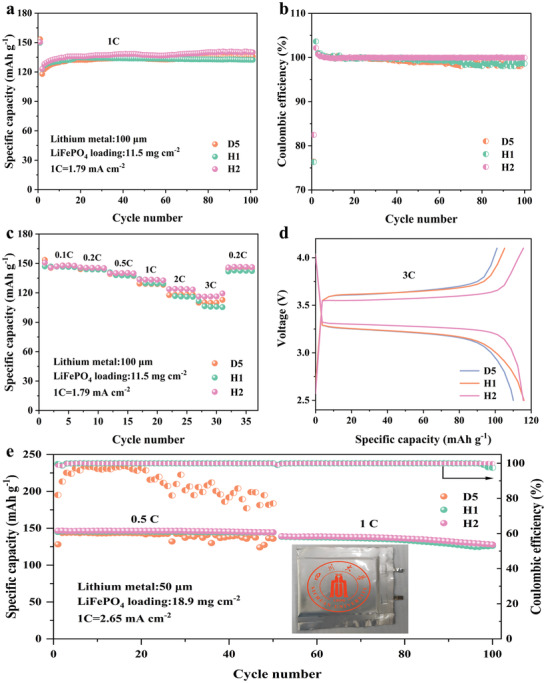
a) Cycling performance and b) corresponding CE of LiIILiFePO_4_ cells cycled in D5, H1, and H2 between 2.5 and 4.1 V at 1 C. c) Rate performance of LiIILiFePO_4_ cells using D5, H1, and H2. d) Voltage profiles of LiIILiFePO_4_ cells using D5, H1, and H2 at 3 C. e) Cycling performance of LiIILiFePO_4_ pouch cells cycled in D5, H1, and H2.

The electrolyte oxidation potential of a LMB matched with a LiFePO_4_ cathode must reach ≈4 V. Figure S11 (Supporting Information) confirms the feasibility of matching D5 and H1 with LiFePO_4_ in a full battery, as the LiIICu battery assembled by H1 shows excellent CE, and its symmetrical battery shows stable cycling stability. For a full cell with H1, TTE is added to dilute H2, and the electrochemical performance of the LiIILiFePO_4_ battery in the D5, H1, and H2 electrolytes is evaluated. We also evaluate the molecular state in the further‐diluted electrolyte H2 using molecular simulations, and the results (Figure S12, Supporting Information) are consistent with those of H1. As shown in Figure S13 (Supporting Information), the CE in the LiIICu cells and the cycling stability of LiIILi batteries with electrolyte H2 both indicate that the concentration still has excellent application prospects after further dilution. The cycle performance in **Figure** **6**a demonstrates that there is a subtle difference in the stability of the battery in the D5, H1, and H2 electrolytes. LMBs with these electrolytes can cycle stably for 100 cycles at a current density of 1 C without capacity attenuation. Importantly, the LMB with H2 displays the highest capacity of 139 mAh g^–1^ at 1 C.

To better understand the electrochemical performance of the full cell, we evaluated the reversibility of the battery in each electrolyte system based on the CE, as shown in Figure 6b. The CE of the full cell in these three electrolytes (D5, H1, and H2) is close to 100%, indicating that D5, H1, and H2 are highly compatible with LiFePO_4_ in coin full cells. The results in Figure 6c show that the rate performance of the battery with H2 is still the best. When the current density suddenly decreases from 3 to 0.2 C, the capacity of the battery can be restored from a specific capacity of 117 to 145 mAh g^–1^. Because H2 exhibits the best rate performance, the charge–discharge voltage profiles of these electrolytes are evaluated at 3 C in Figure 6d. The results indicate that the capacity increases with decreasing apparent concentration of electrolyte owing to the smaller polarization, and Figure S14 (Supporting Information) demonstrates that the better performance with H2 is due to its minimum interfacial resistance. Then, we further increased the content of the TTE diluent and diluted the electrolyte from H2 to H3, and the molecular state was evaluated based on molecular simulations; the results (Figure S15, Supporting Information) are consistent with those for H2. The performance of the half‐cell in the H3 electrolyte is excellent (Figure S16, Supporting Information); however, the life of the full cell is poor owing to the large amount of diluent added (Figure S17, Supporting Information). To evaluate the real application value of H2, the assembled pouch cell shows that H2 has excellent cycling stability (capacity retention of 91.4% at 1 C). As demonstrated in Figure 6e, unlike the coin cell, the electrochemical performance of D5 in the pouch cell is very poor. We believe that the large area of the separator in the pouch cell and the large contact angle with the high‐concentration electrolyte reduce the ion permeability. This type of electrolyte presents good prospects for compatibility with LiFePO_4_. Hence, this electrolyte system can effectively achieve the dual goals of concentration dilution and high availability.

## 3 Conclusions

This study confirms that a LiIICu battery with a diluted high‐concentration electrolyte prepared with LiFSI, THF, and TTE can achieve a CE of ≈99% and stable cycling for 1000 cycles at 1 mA cm^–2^ and 1 mAh cm^–2^. Even when the current density and areal capacity are increased, a CE of 98% can be maintained and is stable for more than 200 cycles. Experimental and theoretical analyses show that the addition of the TTE diluent not only maintains a high CE, but also significantly restrains the high viscosity and poor wettability of high‐concentration electrolytes while realizing a low electrolyte cost. This diluted high‐concentration electrolyte generates a LiF‐rich SEI, which can enhance the CE of the battery considerably. As the electrolyte is further diluted, the LiIILiFePO_4_ exhibits excellent cyclability at 1 C. In this study, the proposed electrolyte system can maintain the high‐concentration effect of the electrolyte while effectively reducing the concentration of the electrolyte. Matched LMBs exhibit excellent electrochemical performance, which opens a new avenue for the commercialization of electrolytes.

## Conflict of Interest

The authors declare no conflict of interest.

## Supporting information

Supporting Information

## Data Availability

The data that support the findings of this study are available from the corresponding author upon reasonable request.
