# Supplementary material for: Ultra‐Low Concentration Electrolyte Enabling LiF‐Rich SEI and Dense Plating/Stripping Processes for Lithium Metal Batteries
Source: Adv Sci (Weinh). 2022 Aug 17;9(28):2203216. doi: 10.1002/advs.202203216 (PMC9534938; doi:10.1002/advs.202203216)
Supplement: Supplementary file 1 — Supporting Information [file ADVS-9-2203216-s001.pdf]

ADVANCED SCIENCE
Open Access

Supporting Information

for *Adv. Sci.*, DOI 10.1002/advs.202203216

Ultra-Low Concentration Electrolyte Enabling LiF-Rich SEI and Dense Plating/Stripping Processes for Lithium Metal Batteries

Ting Chen, Jinhai You, Rong Li, Haoyu Li, Yuan Wang, Chen Wu, Yan Sun, Liu Yang, Zhengcheng Ye, Benhe Zhong, Zhenguo Wu and Xiaodong Guo**

Supporting Information

Ultra-low Concentration Electrolyte Enabling LiF-rich SEI and Dense Plating/Stripping Processes for Lithium Metal Batteries

Ting Chen[a], *Jinhai You*[b], *Rong Li*[a], *Haoyu Li*[a], *Yuan Wang*[a], *Chen Wu*[a], *Yan Sun*[c], *Liu Yang*[d], *Zhengcheng Ye*[a], *Benhe Zhong*[a], *Zhenguo Wu**[a], *and Xiaodong Guo**[a][e]

[a] Department of Chemical Engineering, Sichuan University, Chengdu 610065, PR (China)

[b] Laboratory for Soft Matter and Biophysics, Department of Physics and Astronomy, KU Leuven, Leuven 3001, Belgium

[c] Chengdu University, Chengdu, 610106, PR (China)

[d] School of Materials Science and Engineering, Henan Normal University, Xinxiang, Henan 453007 PR (China)

[e] Institute for Advanced Study, Chengdu University, Chengdu, 610106, PR (China)

*Corresponding authors.

E-mail addresses: zhenguowu@scu.edu.cn (Zhenguo Wu), xiaodong2009@scu.edu.cn (Xiaodong Guo).

Keywords: electrolyte concentration, lithium metal batteries, dense plating/stripping process, solid electrolyte interphase, pouch cell

1. Experimental section

1.1. Materials preparation

Battery-grade THF, LiFSI (99.9%), and TTE (1,1,2,2-Tetrafluoroethyl-2,2,3,3-tetrafluoropropylether, 99.9%) were purchased from Canrd and further dried with molecular sieves overnight before using. The electrolyte is composed of different components according to the molar ratio，refer to the Table S1. The $LiFePO_4$ cathode material purchased from Canrd. The area of the $LiFePO_4$ cathode disks was 1.54 cm^2, and the material loading was approximately 1.79 mAh g^{-1}.

1.2 Electrochemical measurements

Electrochemical cycling tests were carried out using CR2025-type coin cells, and 80μL of electrolyte was added to each coin cell for comparison. The coulombic

efficiency of lithium metal was measured in Li‖Cu cells, in which a piece of commercial copper foil, a separator, and a Li chip (100 μm thick, MTI Corporation) were sandwiched together in a cell inside an argon-filled glovebox. Electrochemical test equipment is Neware battery test system (CT-4008T-5V20mA-164, Shenzhen, China). Firstly, a Li layer with a capacity of 4 mAh cm^{-2} was first deposited and then fully stripped to a cutoff voltage of 1 V. In the following cycles, repeatedly depositing a different areal capacity and then fully stripping Li to 1 V. For Li‖Li or Li‖$LiFePO_4$ cells, the Cu foil was replaced by a Li chip or a piece of $LiFePO_4$ cathode. The electrochemical performances of Li‖$LiFePO_4$ cells with different electrolytes were evaluated within a voltage window of 2.5–4.1 V.

1.3 Materials characterizations

The ^{7}Li liquid state NMR spectra of the electrolytes were recorded on Bruker Avance NEO 600. Raman spectra were measured by HORIBA Scientific LabRAM HR Evolution with an exciting laser of 633 nm. The Fourier transform infrared spectrometer analysis (FTIR, Nicolet 670) was performed in the range of 400–4000 cm^{-1}. XPS analysis was performed on a Thermo Scientific K-Alpha. SEM images were collected on TESCAN VEGA3 SBH. LSV was conducted on a CS2350H electrochemical workstation in the voltage window at a scan rate of 0.1 mV s^{-1}.

1.4. Computational details

In this work, a standard molecular mechanics potential model was used with the following functional form:

$$u\left(r^{N}\right)=\sum_{bonds}\frac{k_i}{2}\left(l_i-l_{i,0}\right)^2+\sum_{angles}\frac{k_i}{2}\left(\theta_i-\theta_{i,0}\right)^2+\sum_{torsions}\frac{V_n}{2}\left(1+\cos\left(n\omega-\gamma\right)\right)$$

$$+\sum_{i=1}^{N}\sum_{j=i+1}^{N}\left(4\varepsilon_{ij}\left[\left(\frac{\sigma_{ij}}{r_{ij}}\right)^{12}-\left(\frac{\sigma_{ij}}{r_{ij}}\right)^{6}\right]+\frac{q_i q_j}{r_{ij}}\right)$$

where the first three terms are the bonded interactions, including bond, angle, and torsion interactions, and the second terms are nonbonded interactions, including van der Waals (vdW) and Coulombic interactions. For different kinds of atoms, the Lorentz-Berthelot mix rules were adopted for vdW interactions, which is following the

equation:

$$\sigma_{ij} = \frac{1}{2}(\sigma_{ii} + \sigma_{jj});\ \varepsilon_{ij} = (\varepsilon_{ii} * \varepsilon_{jj})^{1/2}$$

Five systems were constructed and the detailed information of these systems were summarized in Table 1. The initial configurations of these five systems were constructed using the Packmol software [1]. The simulations were performed using GROMACS package (version 2019.3) with the all-atom OPLS (optimized performance for liquid systems) force field [2]. For each system, the steep descent method was used to minimize the energy of the system. Subsequently, molecular dynamics simulations under NPT ensemble at 298 K and 1 atm were performed for 100 ns for each system. LINCS algorithm was applied to constrain the bond lengths of other components [3]. Periodic boundary conditions were applied in all three directions. The temperature was maintained using the V-rescale thermostat algorithm. The cut-off distance for the Lennard-Jones and electrostatic interactions was 1.2 nm. Particle mesh Ewald method [4] was used to calculate the long-range electrostatic interactions. Configurations were visualized using Visual Molecular Dynamics software [5].

2. Supporting Tables and Figures

Table S1. Summary of the simulated systems

System	N (THF)	N (FSI-)	N (TTE)	The length of the box
D1	7000	500	0	99.58
D5	4200	1500	0	98.35
H1	1540	550	1540	98.03
H2	980	350	1960	99.23
H3	700	250	2100	98.91

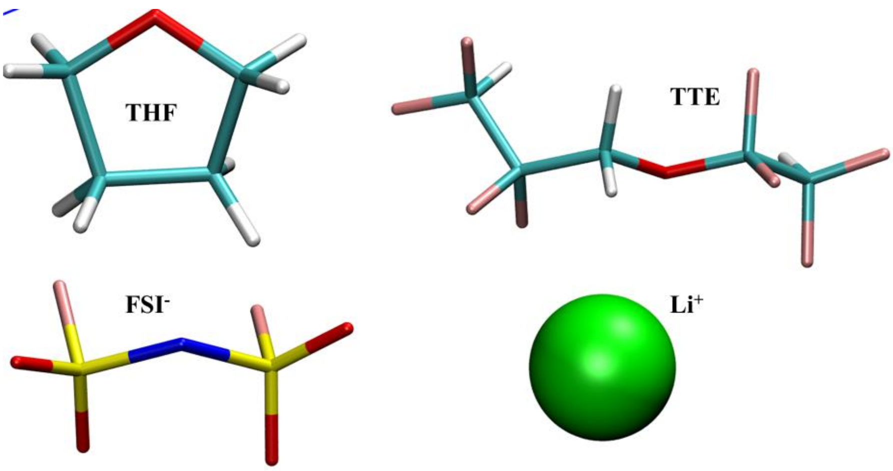

Figure S1. The molecular formula of simulated systems.

Table S2. The ratios of the solvents and the concentration in different electrolytes.

Type / System	Molar ratio (n_{THF}:n_{LiFSI}:n_{TTE})	Concentration (M)
D1	14:1:0	0.991
D3	14:3:0	2.97
D5	14:5:0	4.86
D7	14:7:0	6.93
H1	14:5:14	2.43
H2	14:5:28	1.62
H3	14:5:42	1.22

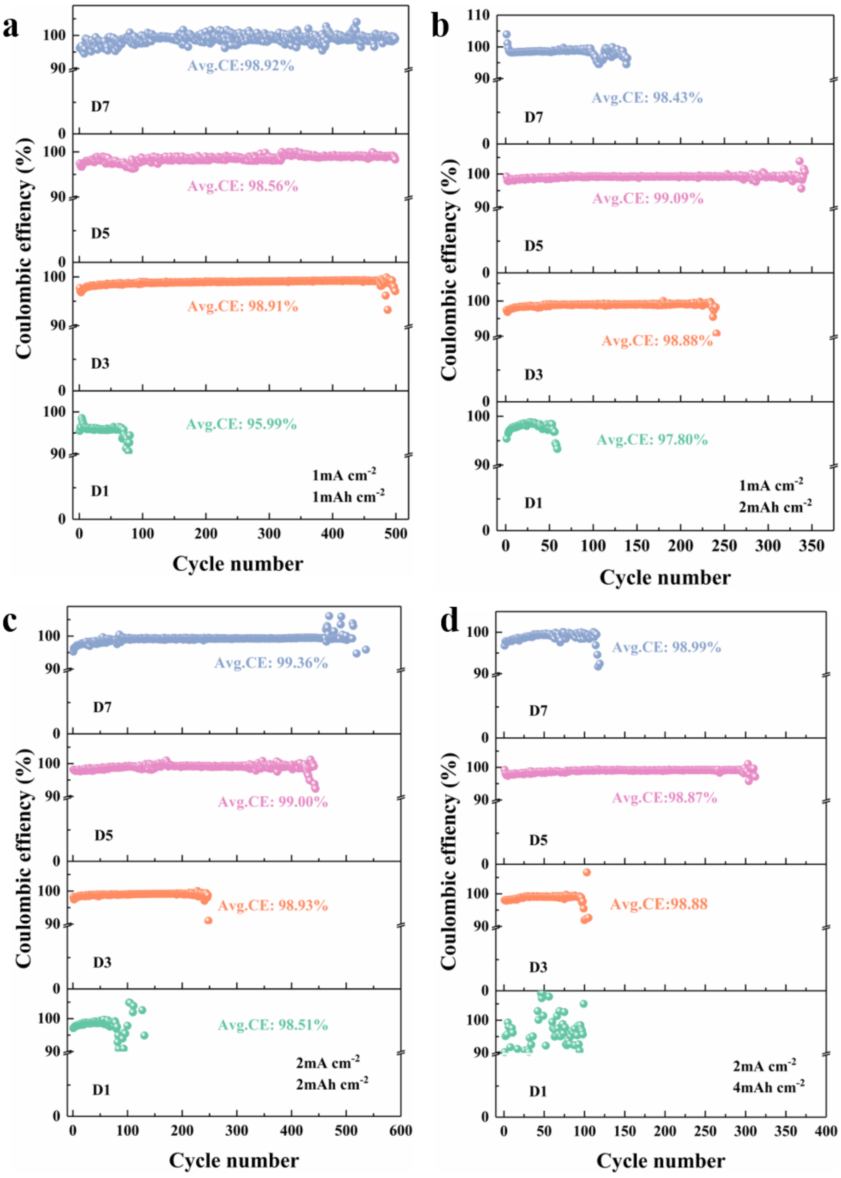

Figure S2. CE of Li‖Cu cells for Li metal on a Cu foil substrate with different current densities.

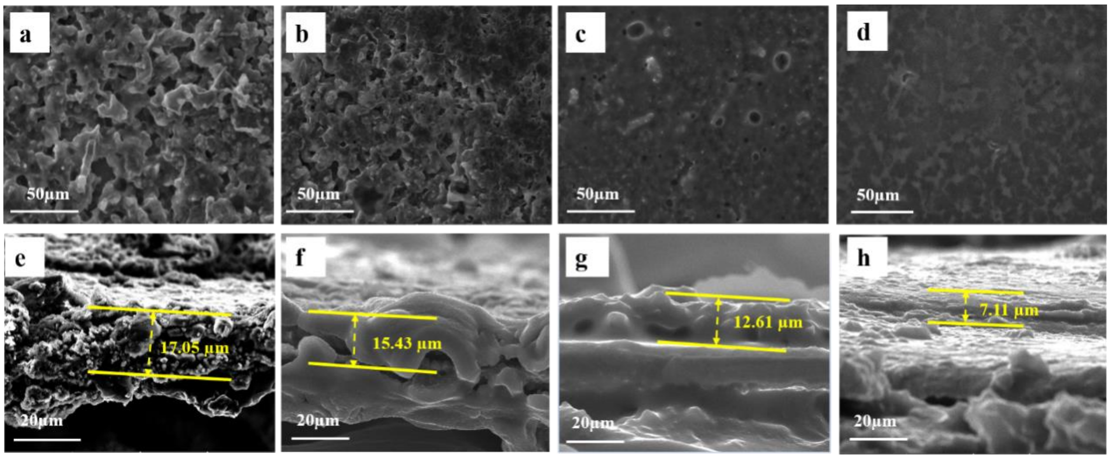

Figure S3. SEM images with top-view and cross-section of Li deposition morphologies in different electrolytes: (a, e) D1, (b, f) D3, (c, g) D5, and (d, h) D7.

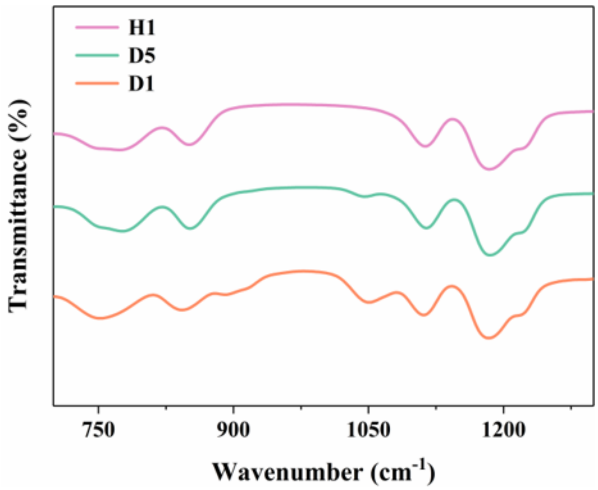

Figure S4. FT-IR characterization results of D1, D5, and H1 at room temperature.

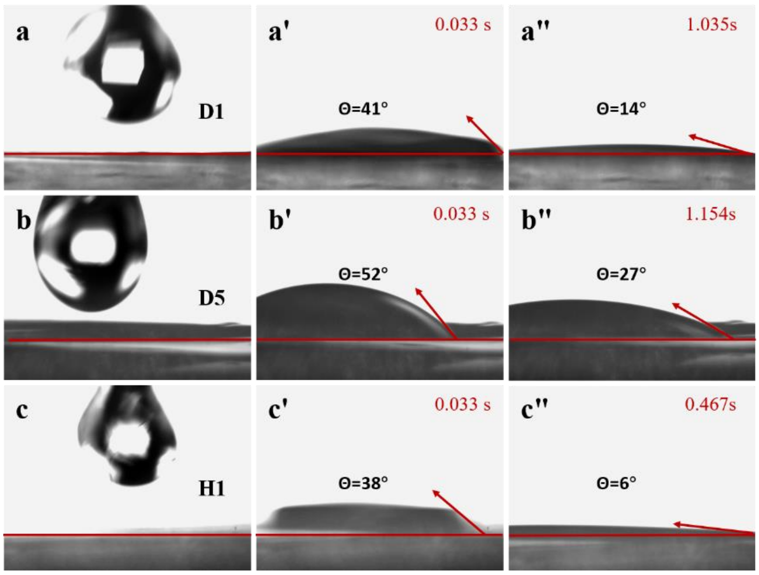

Figure S5. Contact angles between the electrolytes of a) D1, b) D5, and c) H1 with copper foil.

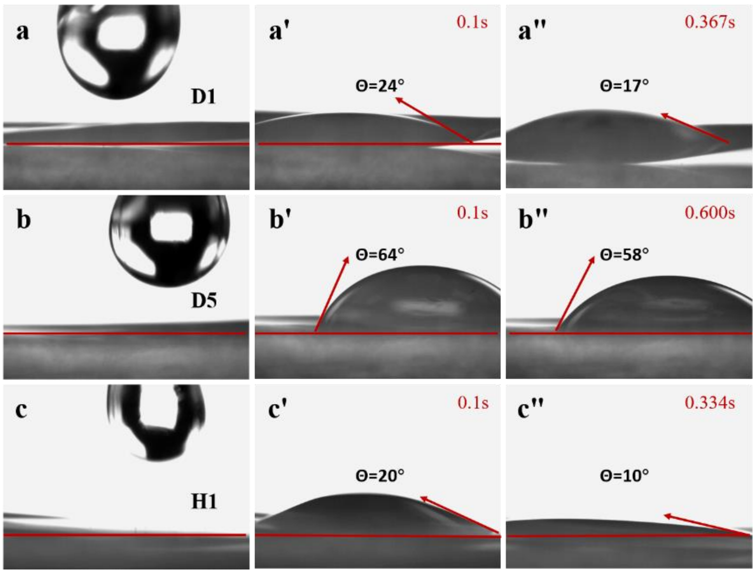

Figure S6. Contact angles between the electrolytes of a) D1, b) D5, and c) H1 with separators.

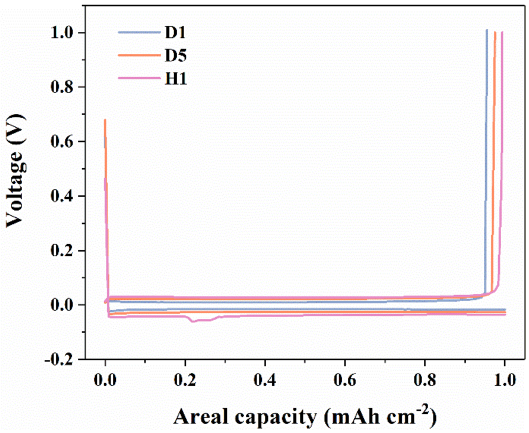

Figure S7. First cycle voltage profiles of Li plating/stripping in different electrolytes at a current density of 0.2 mA cm^{-2} and an area capacity of 1mAh cm^{-2}.

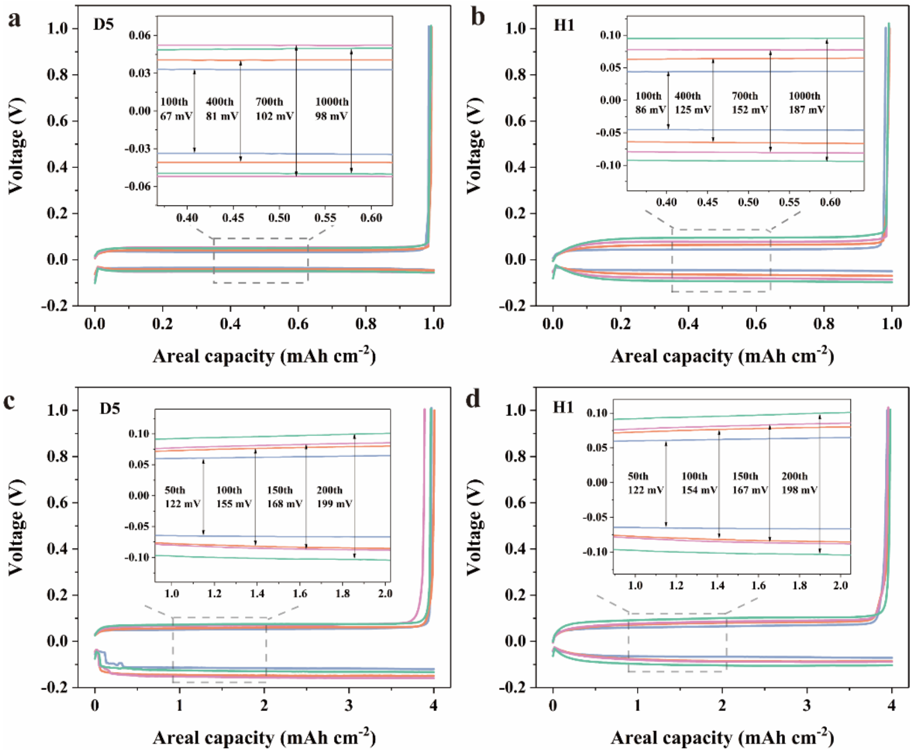

Figure S8. a, b) The plating/stripping profiles in D5 and H1 with 1 mAh cm^{-2} at 1 mA cm^{-2}. c, d) The plating/stripping profiles in D5 and H1 with 4 mAh cm^{-2} at 2 mA cm^{-2}.

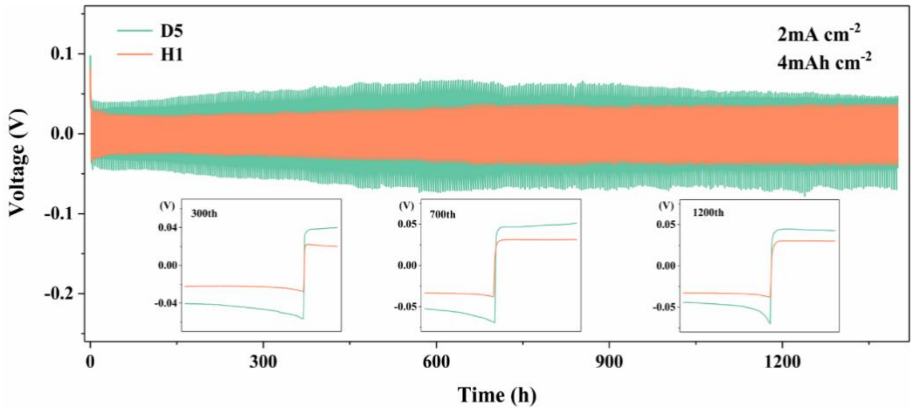

Figure S9. Cycling performance of Li Ⅱ Li symmetrical cells with fixed capacity of 4 mAh cm^{-2} and fixed current density of 2 mA cm^{-2} in D5 and H1.

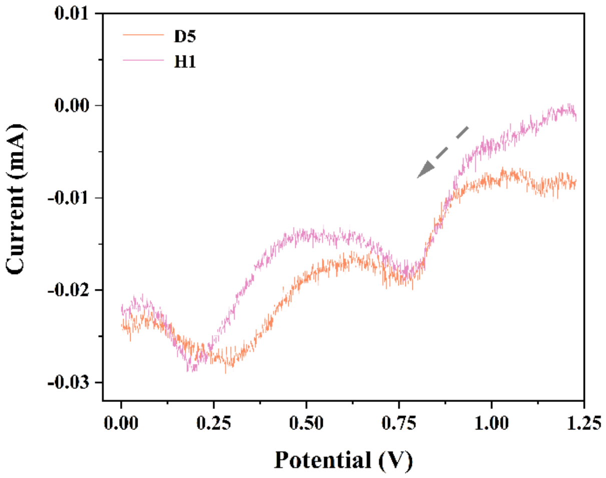

Figure S10. Linear sweep voltammetry (LSV) curves of Cu Ⅱ Li in D5 and H1 electrolytes.

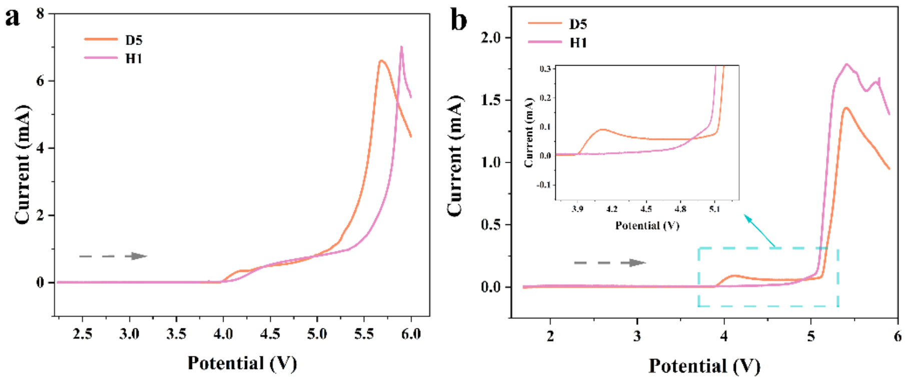

Figure S11. Linear sweep voltammetry (LSV) curves of a) SS Ⅱ Li and b) Al Ⅱ Li cells in D5 and H1 electrolytes (working electrode: Stainless Steel or Al, counter/reference electrodes: Li foil, scanning rate: 1 mV s^{-1}).

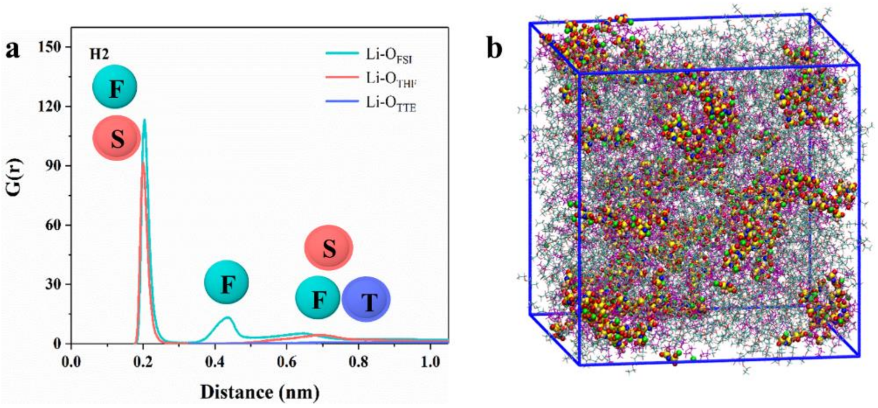

Figure S12. a) Radial distribution function and b) molecular dynamics (MD) simulation results of electrolyte H2.

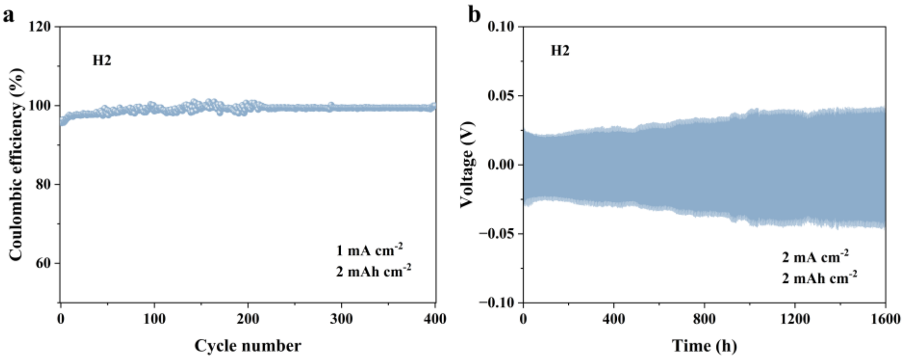

Figure S13. Cycling performance of a) LiⅡCu and b) LiⅡLi symmetrical cells with fixed capacity of 2 mAh cm^{-2} in H2.

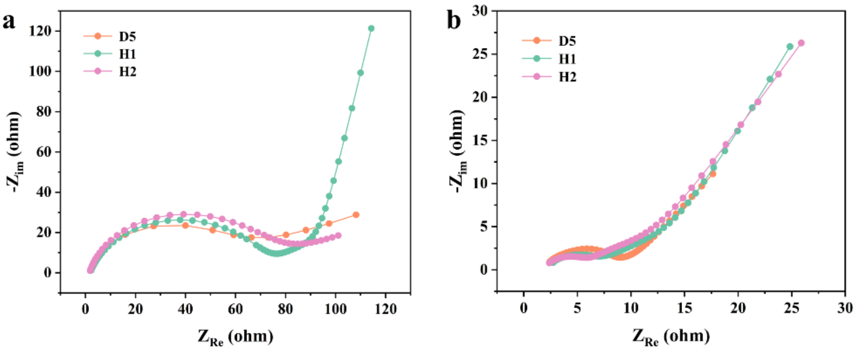

Figure S14. The evolution of impedance spectra of the LiⅡLiFePO$_4$ cells with resting time (a) and after 30th cycles (b) in D5, H1, and H2 electrolytes.

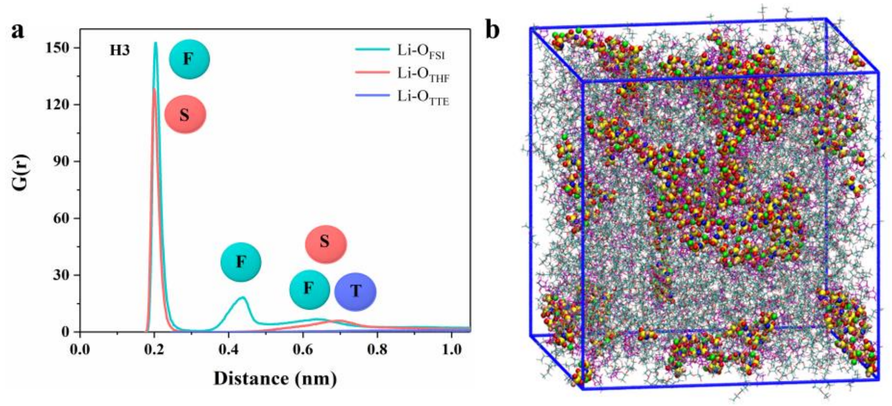

Figure S15. a) Radial distribution function and b) molecular dynamics (MD) simulation results of electrolyte H3.

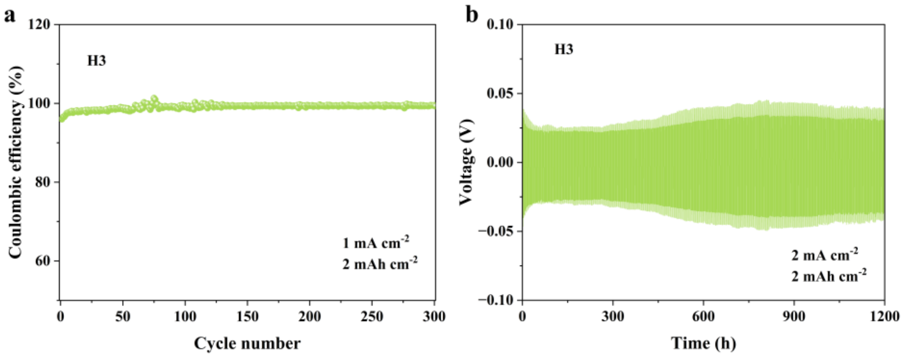

Figure S16. Cycling performance of a) LiⅡCu and b) LiⅡLi symmetrical cells with fixed capacity of 2 mAh cm^{-2} in H3.

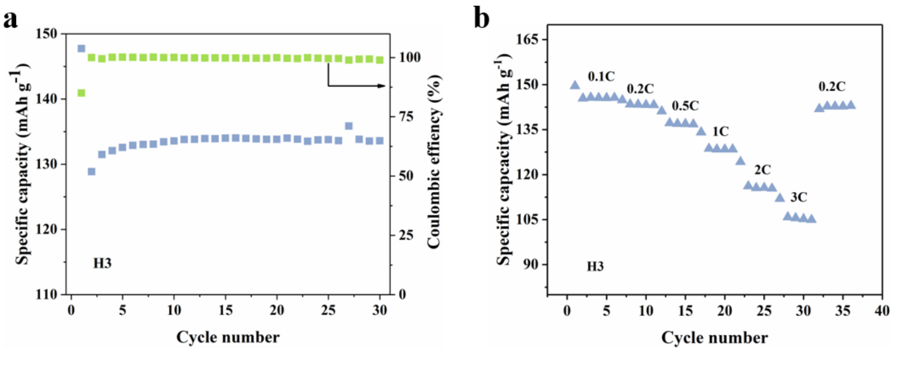

Figure S17. Cycling (a) and rate performance (b) of LiⅡLiFePO$_4$ cells cycled in H3.

References

[1] L. Martínez, R. Andrade, E. G. Birgin, J. M. Martínez, *J. Comput. Chem.* **2009**, *30*, 2157.

[2] M.J. Abraham, T. Murtola, R. Schulz, S. Páll, J.C. Smith, B. Hess, E. Lindahl, *SoftwareX*. **2015**, *6*, 19.

[3] B. Hess, H. Bekker, H. J. C. Berendsen and J. G. E. M. Fraaije, *J. Comput. Chem.* **1997**, *18*, 1463.

[4] G. Bussi, D. Donadio, M. Parrinello, *J. Phys. Chem.* **2007**, *126*, 014101.

[5] W. Humphrey, A. Dalke, K. Schulten, *J. Mol. Graph.* **1996**, *14*, 33.
